# Food Environment index is inversely associated with cancer survival in North Carolina

**DOI:** 10.1371/journal.pone.0326597

**Published:** 2025-07-10

**Authors:** Shenghui Wu, Sarah Ulrich, Tinghao Feng, Yanning Liu, Leilani Tseng

**Affiliations:** 1 Department of Public Health, Beaver College of Health Sciences, Appalachian State University, Boone, North Carolina,; 2 Department of Geography and Planning, Appalachian State University, Boone, North Carolina,; 3 Department of Computer Science, Appalachian State University, Boone, North Carolina,; 4 Department of Psychology & Neuroscience, The University of North Carolina at Chapel Hill, Chapel Hill, North Carolina; Xi’an Jiaotong University, CHINA

## Abstract

**Background:**

Exploring the potential role of the food environment on cancer survival could inform prevention and control strategies to improve cancer survivorship. This study examined the associations between the food environment index (FEI) and survival for overall cancer in North Carolina.

**Methods:**

This retrospective cohort study included 1,292,991 patients diagnosed with overall cancer from January 1, 2000, to December 31, 2022, from the North Carolina Cancer Central Registry. Individual-level demographic, tumor, and treatment data were provided by the North Carolina Cancer Central Registry. FEI and other county-level exposures were obtained from the County Health Rankings & Roadmaps databases. The county-level food environment index measures access to healthy foods (0 is worst, 10 is best). Cox regression was used to calculate hazard ratios (HRs) and 95% confidence intervals (CIs) for the associations between FEI and cancer survival, adjusting for individual-level and county-level covariates.

**Results:**

Higher levels of FEI were associated with statistically significant better survival for overall cancer. The fully adjusted HR for every unit increase in FEI score was 0.983 (95% CI: 0.979, 0.988; *P* < 0.0001). The adjusted HR for the 3^rd^ quartile vs. 1^st^ quartile was 0.98 (95% CI: 0.94, 0.99), and for the 4^th^ quartile vs. 1^st^ quartile it was 0.95 (95% CI: 0.94, 0.96).

**Conclusion:**

These results suggest that a healthy food environment, as measured by FEI, may be a protective factor for cancer survival in North Carolina. To improve cancer survival, further strategies to enhance the food environment at the county level are warranted.

## Introduction

As a leading cause of death in North Carolina [1], cancer is one of the most expensive diseases to manage [2]. Research has shown that cancer risk/survival may be associated with lower levels of healthy dietary intake, including fruits and vegetables [3–8], and whole grains [5,6,8], as well as higher levels of unhealthy dietary intake, such as red meat [5,8,9], processed meat [5,8–10], and excessive alcohol [3,7,8,11]. Geographic variation in incidence and mortality suggests that environmental exposures may play a role in cancer etiology and cancer survival [12–16]. Food environment is an important factor that may impact cancer risk and disease processes, help identify vulnerable populations, and generate results with translational impact relevant to interventionists and policymakers [17]. However, no epidemiologic studies, either globally or in North Carolina have investigated the associations between the food environment and overall cancer survival/risk. To address geographic variations in cancer survival rates and the limited research on the potential role of the food environment, this study aims to examine associations between county-level food environment, as measured by the food environment index (FEI), and overall cancer survival rates in North Carolina using the best available population-based de-identified data.

## Materials and methods

### Research design

This study is a population-based retrospective cohort study using de-identified, limited-use data from the North Carolina Central Cancer Registry. The data were accessed on November 29, 2023. The study did not require informed consent and was exempted from review by the Appalachian State University Institutional Review Board.

### Cancer survival data

Total cancer and death cases for overall cancer identified from the North Carolina Central Cancer Registry were requested and included in the analysis. The North Carolina Central Cancer Registry is a population-based registry that participates in the National Program of Cancer Registries and adheres to data standards established by the North American Association of Central Cancer Registries [18]. To protect patient confidentiality, the North Carolina Central Cancer Registry databases do not include personal identifiers. Data on survival in months, vital status (alive or dead), and cause of death from 100 counties in North Carolina were used for analysis.

### Classification of malignancies

Patients were identified according to the Site Recode International Classification of Disease for Oncology (ICD-O-3)/WHO 2008 Definition, a recoded variable based on site/histology that was provided by the North American Association of Central Cancer Registries [19].

### Patient identification

Inclusion criteria consisted of individuals residing in areas covered by the cancer registries and diagnosed with incident-confirmed cancer. The study included diagnoses made between January 1, 2000, and December 31, 2022, excluding cases reported via autopsy or death certificate only. Survival was defined as the time from initial diagnosis to the time of death, with censoring at the date of last contact or December 31, 2022, whichever occurred first. The North Carolina Central Cancer Registry collects death certificate information, including dates and underlying causes of death, from the state Vital Statistics and the National Death Index to ensure complete and accurate death information, including deaths that occur out of state.

### Data source and measurement for potential factors

Individual-level data on age at diagnosis, sex, race, ethnicity, year of diagnosis, and tumor clinical and treatment information were obtained from the North Carolina Central Cancer Registry. The FEI was sourced from the County Health Rankings & Roadmaps database (2010) [20]. The FEI is an indicator of access to healthy foods, where 0 represents the worst, and 10 represents the best. It equally weighs two indicators of the food environment: 1) limited access to healthy foods: this estimates the percentage of the population that is low-income and does not live close to a grocery store. Low income is defined as having an annual family income of less than or equal to 200 percent of the federal poverty threshold for the family size. “Living close to a grocery store” is defined differently for rural and nonrural areas; in rural areas, it means living less than 10 miles from a grocery store, whereas in nonrural areas, it means living less than 1 mile; and 2) food insecurity: this estimates the percentage of the population that did not have access to a reliable source of food during the past year. A two-stage fixed effects model to estimate food insecurity was created using data from the Community Population Survey, Bureau of Labor Statistics, and American Community Survey [20].

Based on data availability, additional US aggregate county-level data for the year 2000 (or the earliest available year) were obtained from the County Health Rankings & Roadmaps database [21]. These data include measures such as median household income, educational attainment (high school graduation rate), excessive alcohol consumption (average > 1 drink per day for women, or > 2 drinks per day for men), smoking (currently smoke every day or most days and have smoked at least 100 cigarettes in their lifetime), obesity (body mass index (BMI) ≥30 kg/m^2^), and physical inactivity (no leisure time physical activity in the past month). The County Health Rankings & Roadmaps dataset includes 35 county-level measures for 3,200 counties from various national data sources, such as the Behavioral Risk Factor Surveillance System survey, the American Community Survey, the National Center for Health Statistics, and the Centers for Disease Control and Prevention [21]. Counties included in the dataset are assigned Federal Information Processing Standard codes, which were used to compile county-level data.

### Statistical analysis

All county-level data were compiled using unique US Federal Information Processing Standard codes. Chi-square tests for categorical variables and Student t-tests/Wilcoxon Rank-sum test for continuous variables were conducted to assess differences between groups as appropriate. Cox proportional hazard models were used to calculate hazard ratios (HRs) and 95% confidence intervals (CIs) to examine the associations between potential factors and survival time for cancer patients in North Carolina, controlling for covariates. Covariates included individual-level age at diagnosis, sex, race, ethnicity, treatment administration and tumor stage, and year of diagnosis, as well as county-level measures like median household income, high school graduation rate, and the prevalence of excessive alcohol consumption, smoking, obesity, and physical inactivity. These factors were included regardless of their individual significance, to account for their potential impacts on cancer survival. To examine whether a factor modifies the relationship between FEI and cancer survival, interaction terms were added to the model, and likelihood ratio tests were conducted to access statistical significance. All available factors were tested for interactions, and stratified analyses were conducted for significant interactions. Kaplan-Meier survival curves were created to visualize survival probability over time by different subgroups, including gender, race/ethnicity, and age at diagnosis. Additionally, a restricted cubic spline Cox regression was used to evaluate the association of HRs with FEI on a continuous scale after adjusting for other covariates. All statistical tests were two-sided, and a p-value of less than 0.05 was considered statistically significant. Statistical modeling was performed using SAS 9.4 (SAS Institute, Cary, North Carolina). Geographic maps were conducted using JavaScript and ArcGIS Pro (3.4.1).

## Results and discussion

A total of 1,292,991 patients diagnosed with overall cancer between 2000 and 2022 in North Carolina were included in the analysis (Table 1). The median survival time was 9.5 months (interquartile range: 4.2 to 35.1 months). The average age at diagnosis was 64.3 years (standard deviation: 14.7 years), with living survivors having an average age of 59.26 (SD: 14.62 years), and deceased patients having an average age of 69.33 (SD: 12.86 years)f. Female patients comprised 51% of the cohort, and the majority of patients (96.7%) were non-Hispanic patients while Hispanic patients represented 1.6% of the sample. At the end of the follow-up period, 642,389 (49.68%) patients were alive, and 650,602 (50.32%) had deceased. Deceased patients were more likely to be male, older, White, and non-Hispanic They were also more likely to have “regional or distant” tumor stages. reside in counties with lower FEI scores, live in areas with lower median household income, and have lower high school graduation rates Additionally, deceased patients were more likely to live in counties with higher percentages of adults who were obese, smoked, were physical inactive, or engaged in excessive alcohol consumption (all *P*s < 0.05). The most common cancer types in North Carolina were female breast, lung, prostate, skin, colorectal, and bladder cancers.

**Table 1:** Characteristics by the Vital Status among North Carolina Cancer Patients.

Characteristics	Vital status (No, %)			*P* value
	Dead (n = 642,389)	Alive (n = 650,602)	Total (1,292,991)	
Individual-level data				
Gender				<.0001
Female	298,939 (46.54)	361,527 (55.57)	660,466	
Male	343,420 (53.46)	289,007 (44.42)	632,427	
Other	30 (0.01)	68 (0.01)	98	
Race				<.0001
White	506,772 (78.89)	516107 (79.33)	1,022,879	
Black	124,074 (19.31)	110946 (17.05)	235,020	
Asian or Pacific Islander	4,002 (0.62)	7856 (1.21)	11,858	
American Indian or Alaskan Native	5,115 (0.80)	4373 (0.67)	9,488	
Other	2,426 (0.38)	11,320 (1.74)	13,746	
Ethnicity				<0.0001
Hispanic	5,991 (0.93)	14,881 (2.29)	20,872	
Non-Hispanic	624,567 (97.23)	625,689 (96.17)	1,250,256	
Others	11,831 (1.84)	10,032 (1.54)	21,863	
Stage^c^				<0.0001
In situ or localized	222,357 (34.61)	447,257 (68.75)	669,614	
Regional or distant	339,891 (52.91)	169,826 (26.10)	509,717	
Unknown	80,141 (12.48)	33,519 (5.15)	113,660	
Treatment				<0.0001
Yes	500466 (77.91)	587,943 (90.37)	1,088,409	
No	51,109 (7.96)	21,517 (3.31)	72,626	
Unknown	90,814 (14.14)	41,142 (6.32)	131,956	
Surgery				<0.0001
Yes	324,324 (50.49)	504,480 (77.54)	828,804	
No	297,440 (46.30)	144,898 (22.27)	442,338	
Unknown	21,133 (3.15)	1,224 (0.19)	21,849	
Chemotherapy				<0.0001
Yes	213,778 (33.28)	147,172 (22.62)	360,950	
No	400,185 (62.30)	497,313 (76.44)	925,146	
Unknown	28,426 (4.43)	6,117 (0.94)	34,958	
Radiation therapy				<0.0001
Yes	175,325 (27.29)	175,786 (27.02)	351,111	
No	427,331 (66.52)	459,166 (70.58)	897,498	
Unknown	39,733 (6.19)	15,650 (2.41)	34,543	
Diagnosis age (years; mean ± SD)	69.33 (12.86)	59.26 (14.62)	64.26 (14.67)	<0.0001
Cancer site				<0.0001
Female breast	63,016 (9.81)	147,666 (22.70)	210,682	
Lung/bronchus	139,927 (21.78)	27,171 (4.18)	167,098	
Prostate	55,784 (8.68)	99,838 (15.35)	155,622	
Melanoma (skin)	25,277 (3.93)	84,614 (13.01)	109,891	
Colon/rectum	59,843 (9.30)	41,561 (6.39)	101,404	
Bladder	26,386 (4.11)	20,563 (3.16)	46,949	
Brain/other CNS	19,480 (3.03)	24,209 (3.71)	43,689	
Non-Hodgkin’s lymphoma	22,156 (3.45)	20,356 (3.13)	42,512	
Kidney	19,294 (3.00)	22,482 (3.46)	41,776	
Endocrine	6,310 (0.98)	29,096 (4.47)	35,406	
Corpus uteri	12,055 (1.88)	20,919 (3.22)	32,974	
Leukemia	18,773 (2.92)	14,001 (2.15)	32,774	
Oral cavity	16,770 (2.61)	13,806 (2.12)	30,576	
Pancreas	27,015 (4.21)	3,346 (0.51)	30,361	
Multiple myeloma	11,493 (1.79)	6,656 (1.02)	18,149	
Liver	15,003 (2.34)	2,850 (0.44)	17,853	
Stomach	11,000 (1.71)	4,018 (0.62)	15,018	
Ovary	9,344 (1.45)	5,571 (0.86)	14,915	
Cervix uteri	3,930 (0.61)	7,419 (1.14)	11,349	
Esophagus	9,335 (1.45)	1,767 (0.27)	11,102	
Larynx	6,897 (1.07)	3,963 (0.61)	10,860	
Soft tissue	3,845 (0.60)	3,622 (0.56)	7,467	
Hodgkins’s disease	1,464 (0.23)	4,213 (0.65)	5,677	
Testes	543 (0.08)	4,560 (0.70)	5,103	
Gallbladder	2,082 (0.32)	370 (0.06)	2,452	
Bone	924 (0.14)	1,111 (0.17)	2,035	
Other cancers	54,443 (8.48)	34,854 (5.36)	89,297	
County-level data				
Food environment index (median, interquartile range)	6.80 (1.04)	6.85 (1.06)	6.82 (1.08)	<0.0001
Median household income				
Low (≤$43,299)	360,009 (56.04)	317,012 (48.73)	677,021	
High (>$43,299)	282,380 (43.96)	333,590 (51.27)	615,970	
High school graduation rate				<0.0001
Low (<76%)	222,956 (34.75)	217,920 (33.53)	440,876	
Medium (76%−81.2%)	219,050 (34.14)	202,091 (31.09)	421,141	
High (>81.2%)	199,538 (31.10)	229,992 (35.38)	429,530	
Percent of adults that reported currently smoking				<0.0001
Low (<17.4%)	192,434 (30.33)	242,168 (37.57)	434,602	
Medium (17.4%−22.8%)	214,701 (33.84)	207,343 (32.17)	422,044	
High (>22.8%)	227,361 (35.83)	195,066 (30.26)	422,427	
Percent of adults with BMI ≥ 30 kg/m^2^				
Low (≤28.5%)	306,365 (47.69)	356,456 (54.76)	662,821	<.0001
High (>28.5%)	336,024 (52.31)	294,321 (45.24)	630,170	
Percent of adults that report no leisure time physical activity				<.0001
Low (≤25.6%)	294,771 (45.89)	356,281 (54.75)	651,052	
High (>25.6%)	347,618 (54.11)	298,024 (45.25)	641,939	
Percent of adults that report excessive alcohol drinking				
Low (<12.3%)	199,592 (34.96)	172,952 (29.08)	372,544	<.0001
Medium (12.3%−15%)	228,866 (40.09)	261,387 (43.95)	490,253	
High (>15%)	142,441 (48.98)	160,400 (26.97)	302,841	

BMI: body mass index.

CNS: central nervous system.

**Fig 1:**
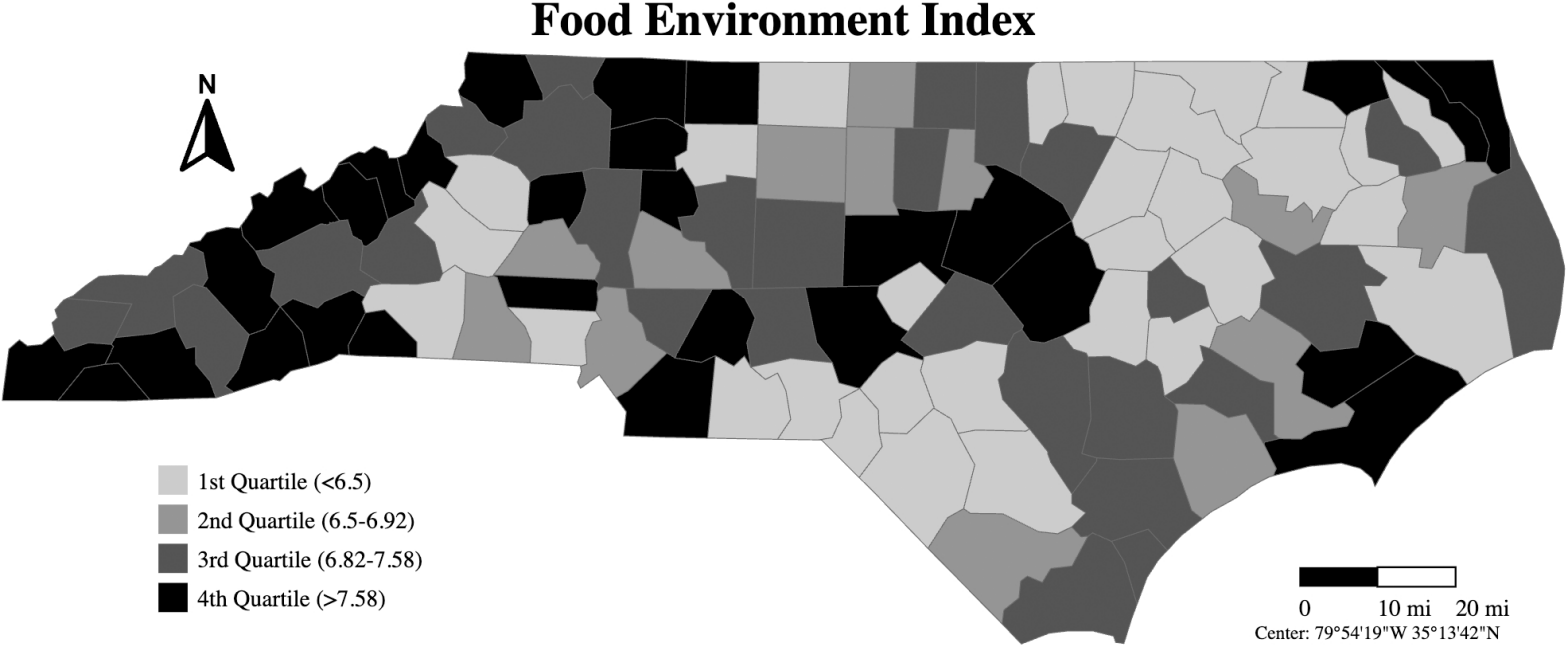
Food Environmental Index Across 100 Counties in the North Carolina Cancer Registry Catchment Areas.

**Fig 2:**
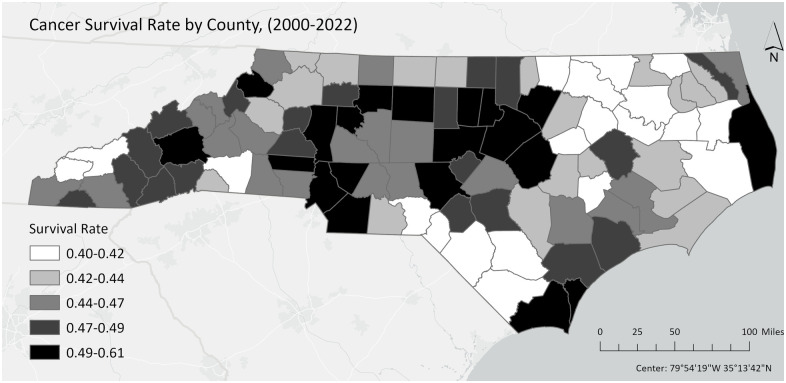
Survival Rates for Overall Cancer Across 100 Counties in the North Carolina Cancer Registry Catchment Areas.

**Fig 1** shows FEI scores categorized by quartiles calculated using data from all 100 counties included in the study. Annual average FEI scores ranged between 4.44 and 8.73. Generally, higher average FEI scores were observed in western (e.g., Madison, Cherokee, Avery, etc.) and northern (e.g., Ashe, Surry, Strokes, etc.) counties of North Carolina. Lower FEI scores were typically found in the northeastern (e.g., Northampton, Halifax, Warren, etc.) and some southern (e.g., Scotland, Robeson, Bladen) counties of North Carolina. Similarly, higher survival rates were observed in western and northern counties, while lower survival rates were noted in the northeastern and some south counties (**Fig 2**).

FEI scores were inversely associated with overall cancer survival in basic models, adjusted for sex, diagnosis age, race, and year of diagnosis (**Table 2**). Every unit increase in the FEI score was associated with a 3.8% reduction in overall cancer survival (adjusted HR for each score increase: 0.962, 95% CI: 0.959, 0.965, *P* < 0.0001). After further adjustment for individual-level ethnicity, treatment administration, tumor stage, and county-level measures of median household income, high school graduation rate, and the prevalence of excessive alcohol consumption, smoking, obesity, and physical inactivity, we still observed a statistically significant inverse association between FEI scores and overall cancer survival (adjusted HR per 1-score increase: 0.983, 95% CI: 0.979, 0.988). Compared to the 1^st^ quartile of FEI scores, FEI scores in the 2^nd^ quartile, 3^rd^ quartile, and 4^th^ quartile categories were inversely associated with overall cancer survival in both the basic and fully adjusted models, except for the fully adjusted model for the 2^nd^ quartile. In contrast to the 1^st^ quartile, the 3^rd^ quartile (HR = 0.98, 95% CI: 0.94, 0.99, *P* = 0.005) and 4^th^ quartile (HR = 0.95, 95% CI: 0.94, 0.96, *P* = 0.001) categories were associated with a 2% and 5% reduced overall cancer survival, after adjustment for other covariates. Statistically significant interactions were observed between FEI and year of diagnosis (*P* for interaction < 0.0001), median household income (*P* < 0.0001), stage (*P* < 0.0001), and obesity percentage (*P* < 0.0001). However, no statistically significant interactions were found for age, sex, race, anatomic site, urbanicity, physical inactivity, excessive alcohol consumption, or region (*Ps* > 0.05). FEI was borderline inversely associated with overall cancer survival in populations diagnosed between 2000–2011 (fully adjusted HR = 0.994, 95% CI 0.988, 1.000), and inversely associated with those diagnosed between 2012–2022 (fully adjusted HR = 0.969, 95% CI 0.962, 0.976) (**Table 3**). FEI was inversely associated with overall cancer survival in areas with high median household income (>$43,299) (adjusted HR = 0.974, 95% CI 0.967, 0.981), and in patients with regional or distant stage (adjusted HR = 0.978, 95% CI 0.972, 0.985). The inverse association between FEI and overall cancer survival was stronger in areas with lower obesity percentage (≤28.5%) (adjusted HR = 0.977, 95% CI 0.970, 0.983) compared to those with a higher obesity percentage (>28.5%) (adjusted HR = 0.984, 95% CI 0.981, 0.987) (**Table 3**).

**Table 2:** Cox Proportional Hazards Regression Analyses of the Associations Between Food Environment Index and Overall Cancer Survival (NC Cancer Registry 2000–2022).

Food environment index	N	Basic adjusted HR^a^	*P*	Fully adjusted HR^b^	*P*
Overall analysis (every score increase)	1,292,991	0.962 (0.959-0.965)	<0.0001	0.983 (0.979-0.988)	<0.0001
FEI quartiles		0.971 (0.969-0.973)	<0.0001	0.984 (0.981-0.987)	<0.0001
First quartile (<6.50)	325,384	Reference		Reference	
Second quartile (6.50–6.82)	329,810	0.915 (0.908-0.921)	<0.0001	0.998 (0.990-1.006)	0.68
Third quartile (6.82–7.58)	313,265	0.963 (0.956-0.970)	<0.0001	0.98 (0.94-0.99)	<0.0001
Forth quartile (>7.58)	324,532	0.893 (0.887-0.900)		0.95 (0.94-0.96)	

HR: hazard ratio.

^a^Adjusted for age at diagnosis, sex, race, and year of diagnosis.

^b^Additionally adjusted for individual-level ethnicity, treatment administration, and tumor stage, and county-level measures of median household income, high school graduation rate, and prevalence of excessive alcohol consumption, smoking, obesity, and physical inactivity.

**Table 3:** Stratified Analysis: Cox Proportional Hazard Model for Associations Between Food Environment Index and Overall Cancer Survival (NC Cancer Registry 2000–2022).

Food environment index	N	Basic adjusted HR^a^	*P*	Fully adjusted HR^b^	*P*
Year of diagnosis					
2000-2011	575,630	0.977 (0.972-0.981)	<0.0001	0.994 (0.988-1.000)	0.06
2012-2022	717,361	0.947 (0.942-0.952)	<0.0001	0.969 (0.962-0.976)	<0.0001
Median household income					
Low (≤$43,299)	677,021	0.997 (0.992-1.001)	0.16	1.000 (0.993-1.007)	0.999
High (>$43,299)	615,970	0.962 (0.956-0.968)	<0.0001	0.974 (0.967-0.981)	<0.0001
Stage					
In situ or localized	669,614	0.979 (0.973-0.985)	<0.0001	1.007 (0.999-1.016)	0.08
Regional or distant	509,717	0.961 (0.956-0.965)	<0.0001	0.978 (0.972-0.985)	<0.0001
Obesity percentage					
Low (≤28.5%)	662,821	0.989 (0.983-0.995)	0.0001	0.977 (0.970-0.983)	<.0001
High (>28.5%)	630,170	0.988 (0.983-0.992)	<0.0001	0.990 (0.983-0.997)	0.005

HR: hazard ratio.

^a^Adjusted for age at diagnosis, sex, race, and year of diagnosis.

^b^Additionally adjusted for individual-level ethnicity, treatment administration, and tumor stage, and county-level measures of median household income, high school graduation rate, and prevalence of excessive alcohol consumption, smoking, obesity, and physical inactivity.

**Fig 3:**
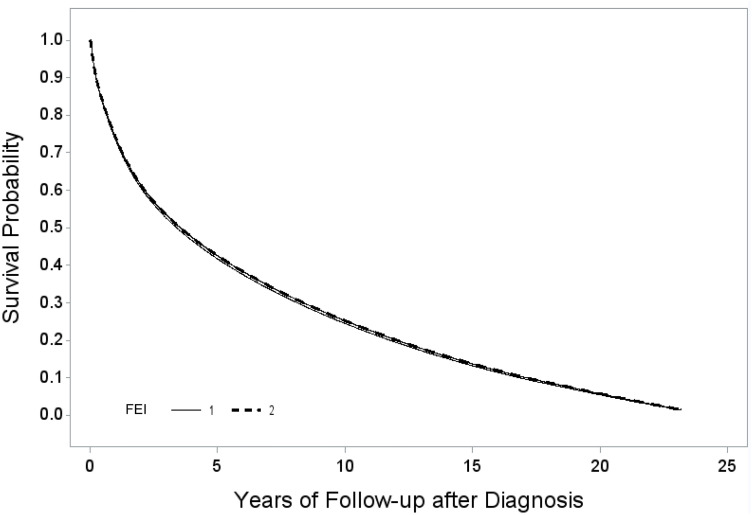
Adjusted* Kaplan-Meier Survival Curves for Overall Cancer North Carolina by Food Environment Index Status. Food environment index (FEI):1: ≤ 6.82; 2: > 6.82. *Adjusted for individual-level age at diagnosis, sex, race, ethnicity, year of diagnosis, treatment administration, and tumor stage, as well as county-level measures, including median household income, high school graduation rate, and the prevalence of excessive alcohol consumption, smoking, obesity, and physical inactivity.

**Fig 3** illustrates the multivariable-adjusted Kapan-Meier survival curves for patients with high and low FEI scores among those with overall cancer. Patients with higher FEI scores had statistically significant better survival compared to those patients with lower FEI scores (*P *< 0.0001).

### Food environment index (FEI):1: ≤ 6.82; 2: > 6.82

*Adjusted for individual-level age at diagnosis, sex, race, ethnicity, year of diagnosis, treatment administration, and tumor stage, as well as county-level measures, including median household income, high school graduation rate, and the prevalence of excessive alcohol consumption, smoking, obesity, and physical inactivity.

**Fig 4:**
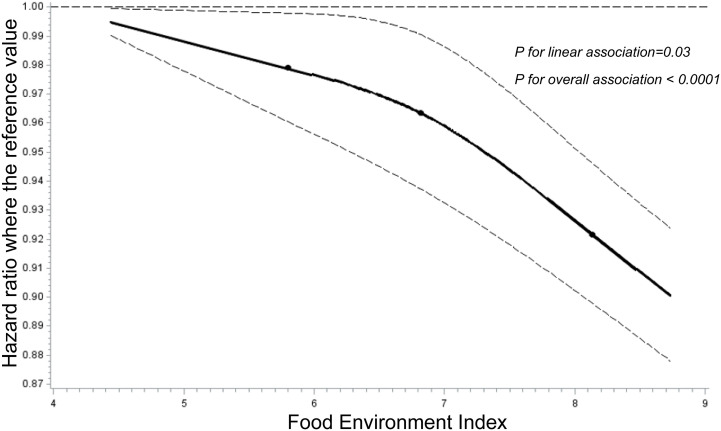
Adjusted Dose-response Association between Food Environment Index (FEI) and Risk for Overall Cancer in North Carolina. FEI was modeled using a restricted cubic spline function with three knots at the 5^th^, 50^th^, and 95^th^ percentiles of FEI distribution. *Y*-axis represents the adjusted hazard ratio for overall cancer death for any given FEI value, compared to those with a FEI of 1. The model was adjusted for individual-level factors, including age at diagnosis, sex, race, ethnicity, year of diagnosis treatment administration, and tumor stage, as well as county-level measures, including median household income, high school graduation rate, and the prevalence of excessive alcohol consumption, smoking, obesity, and physical inactivity. Dashed lines are 95% confidence intervals. Knots are indicated by dots.

**Fig 4** visually depicts the dose-response association between FEI and HRs of overall cancer after adjusting for all potential confounding variables in a restricted cubic spline Cox model. The results show that FEI is inversely associated with the HR for overall cancer (*P* for linear relation = 0.03 and *P* for overall relation < 0.0001).

## Discussion

This study found statistically significant inverse associations between the county-level food environment and overall cancer survival in North Carolina, after adjusting for individual- and county-level demographics, lifestyle, and socioeconomic factors.

Population-based research on the impact of the food environment on cancer risk and survival has been limited. Our previous research identified an inverse association between FEI and gastric cancer incidence in the US [22], but North Carolina data were not included in the US cancer registry. Another study observed a non-significant positive association between an unhealthy food environment and colorectal cancer incidence in a Texas ecological study [23]. Our findings provided the first examination of the inverse association between FEI and cancer survivorship in North Carolina. Additionally, we extended the existing literature by demonstrating that FEI was more strongly associated with overall cancer survivorship in areas with lower obesity percentages. Our results also contribute to the literature by revealing that the FEI-cancer survival association was stronger in more recent years and among patients with higher median household income or with regional/distant tumor stages. Our observation of higher FEI scores and survival rates in western and northern counties of North Carolina (which had higher income levels and better access to grocery stores, improving access to healthy food and food security [20]), and lower FEI scores and survival rates in northeastern and some southern counties (which had lower income and poorer access to healthy food and food security [20]), aligns with the results from the regression models and the dose-response relationship generated by the cubic spline. This may be due to the fact that the food environment, which encompasses both food availability and food access, has been shown to influence healthier dietary attitudes and behaviors [20,24–27]. A higher FEI indicates better access to healthy diet, which may be associated with cancer risk/survival [3–8]. Integrating social and environment factors into cancer survivorship research can enhance our understanding of disease prognosis, identify high-risk populations, and generate insights for further prevention and control efforts [17]. Future research incorporating food environment measures could focus on both community- and individual-level interventions aimed at improving the food environment and dietary behaviors.

Our study has some strengths. First, it utilizes a large sample size of confirmed cancer patients from the North Carolina population-based cancer registry, which encompasses all cancers diagnosed within the state. Second, the study covers all counties in North Carolina, providing a diverse range of FEI scores. Third, the individual- and county-level data on survivorship, clinical and tumor characteristics, and potential confounders and effect modifiers from the North Carolina cancer registry and the County Health Rankings & Roadmaps adds to the comprehensiveness of the study. Finally, as a cohort study, it offers a valuable opportunity to explore the dose-response associations of FEI with cancer survival.

The present study also has several limitations. First, an ecological fallacy may apply, as the association between county-level food environment and cancer survival may not accurately reflect individual-level associations. However, all counties used the same method for assessing the food environment within the study. Second, county-level data may not fully capture individual-level characteristics, which introduces the potential for ecological fallacy [28]. Lastly, residual confounding variables, attributable to lack of information on individual-level factors for cancer survivorship, cannot be ruled out. Nevertheless, we adjusted for individual-level factors such as age, sex, race, ethnicity, year of diagnosis, tumor stage, and treatment administration, as well as county-level variables that may influence cancer survival, including excessive alcohol consumption, smoking, obesity, physical inactivity, and socioeconomic factors.

## Conclusions

Results from the first survival analysis in North Carolina suggest that a healthy food environment, as measured by FEI, may be an important protective factor for overall cancer survival in the state. To enhance cancer survivorship, further strategies to improve the food environment at the county level are recommended.
